# HIV-1 Transactivator Protein Induces ZO-1 and Neprilysin Dysfunction in Brain Endothelial Cells via the Ras Signaling Pathway

**DOI:** 10.1155/2017/3160360

**Published:** 2017-05-02

**Authors:** Wenlin Jiang, Wen Huang, Yanlan Chen, Min Zou, Dingyue Peng, Debing Chen

**Affiliations:** ^1^Department of Neurology, First Affiliated Hospital, Guangxi Medical University, Nanning 530021, China; ^2^Nanxishan Hospital of Guangxi Zhuang Autonomous Region, Guilin 541000, China

## Abstract

Amyloid beta (A*β*) deposition is increased in human immunodeficiency virus-1- (HIV-1-) infected brain, but the mechanisms are not fully understood. The aim of the present study was to evaluate the role of Ras signaling in HIV-1 transactivator protein- (Tat-) induced A*β* accumulation in human cerebral microvascular endothelial cells (HBEC-5i). Cell viability assay showed that 1 *μ*g/mL Tat and 20 *μ*mol/L of the Ras inhibitor farnesylthiosalicylic acid (FTS) had no significant effect on HBEC-5i cell viability after 24 h exposure. Exposure to Tat decreased protein and mRNA levels of zonula occludens- (ZO-) 1 and A*β*-degrading enzyme neprilysin (NEP) in HBEC-5i cells as determined by western blotting and quantitative real-time polymerase chain reaction. Exposure to Tat also increased transendothelial transfer of A*β* and intracellular reactive oxygen species (ROS) levels; however, these effects were attenuated by FTS. Collectively, these results suggest that the Ras signaling pathway is involved in HIV-1 Tat-induced changes in ZO-1 and NEP, as well as A*β* deposition in HBEC-5i cells. FTS partially protects blood-brain barrier (BBB) integrity and inhibits A*β* accumulation.

## 1. Introduction

Human immunodeficiency virus-1- (HIV-1-) related cognitive impairment and dementia are more prevalent in older HIV-1-infected individuals [1, 2]. Amyloid beta (A*β*) deposition is a characteristic in the HIV-1-infected brains [1, 3, 4]. Reportedly, exposure to HIV-1 increases brain A*β* levels by stimulating A*β* formation [5], upregulating amyloid precursor protein expression [6], inhibiting A*β* degradation [7], or altering A*β* transport across the blood-brain barrier (BBB) [8]. The BBB plays a critical role both in HIV-1 trafficking into the brain [9] and in A*β* pathology [1]. The major components of the BBB are brain microvascular endothelial cells [10] joined by tight junctions (TJs) [11]. TJ proteins such as occludin, claudin, and zonula occludens (ZO) play a critical role in maintaining BBB integrity and low permeability [12]. ZO-1 linking the transmembrane TJs (occludin) to actin cytoskeleton plays an important role in barrier resistance and permeability [13, 14].

HIV-1 transactivator of transcription (Tat) protein is essential for HIV infection and virus replication [15]. HIV-1 Tat is involved in several cellular processes including inducing angiogenesis, modulating cytokine expression, and activating cellular signaling pathways [16]. Tat protein is actively secreted by HIV-1-infected cells [10, 15] and may cross the BBB [10] and be taken up by astrocytes and neurons, resulting in neuronal apoptosis and BBB disruption by increasing intracellular calcium and reactive oxygen species (ROS) levels and stimulating inflammation [16]. HIV-1 Tat also inhibits the A*β*-degrading enzyme neprilysin and restricts its ability to degrade A*β*, resulting in increased soluble A*β* levels in cell culture [7]. Cerebral clearance of soluble A*β* involves both degradation in the brain and transport across the BBB to systemic circulation. Neprilysin-degraded A*β* is partially removed from the brain by efflux transport at the BBB [17]. The Ras family of small GTPases transmits extracellular signals involved in many cellular processes that regulate cell growth, differentiation, motility, and death. Ras is a major hub of many signaling pathways [18]. For example, activation of the Ras/mitogen-activated protein kinase pathway is involved in HIV-1 Tat-induced disruption of TJ proteins in brain endothelial cells [19]. Ras signaling induces the generation of ROS via activating the downstream effector mitogen-activated protein kinase (MEK)/extracellular signal-regulated kinases (ERK), resulting in the alteration of TJs and BBB permeability [20]. Farnesylthiosalicylic acid (FTS) is a synthetic and functional Ras inhibitor [21] that has been successfully used in phase II clinical trials of patients with pancreatic and non-small-cell lung cancer [22]. Inhibition of Ras by FTS may ameliorate inflammatory conditions [23]. Ras signaling can be a target for HIV-1 Tat-induced changes of TJ proteins [3], and inhibition may be an appropriate therapeutic intervention [21].

Whether the Ras signaling pathway is involved in HIV-1-induced BBB disruption and A*β* deposition is not fully understood. The aim of the current study was to evaluate the role of Ras signaling in HIV-1-induced ZO-1 and neprilysin disruption and A*β* accumulation in HBEC-5i cells.

## 2. Materials and Methods

### 2.1. Cell Cultures and Treatment

Human cerebral microvascular endothelial cells (HBEC-5i) were purchased from American Type Culture Collection (ATCC, Manassas, VA, USA). The cells were cultured on 0.1% gelatin solution (ATCC)-coated flasks in DMEM:F12 medium (ATCC) supplemented with 10% foetal bovine serum (Gibco/Thermo Fisher, Waltham, MA, USA), 40 *μ*g/mL endothelial growth supplement (ECGS, ATCC), and 1% penicillin-streptomycin (Beyotime, Shanghai) according to the manufacturer's instructions. HBEC-5i cells were incubated at 37°C in a humidified atmosphere of 5% CO_2_.

Recombinant HIV-1 Tat clade-B protein (amino acids 1 to 86) was purchased from Prospec (Rehovot, Israel). The previous literature indicated that concentrations of Tat in HIV-infected patients could reach the range of 0.5 *μ*g/mL of serum [24], so this concentration was used in subsequent experiments. Controls consisted of cells treated with 0.02% DMSO or heat-inactivated Tat (1 *μ*g/mL). Before exposure to 1 *μ*g/mL HIV-1 Tat for 12 or 24 h, confluent HBEC-5i cells were pretreated with 5 *μ*mol/L FTS for 3 h and FTS was retained in the serum-free cell culture medium during Tat treatment as previously described [25].

### 2.2. Cell Viability Assay

A density of 1 × 10^4^ cells/well of HBEC-5i cells were seeded onto 96-well plates. After 12 h, HBEC-5i were treated with Tat at 0, 0.25, 0.5, 1, or 1.25 *μ*g/mL and heat-inactivated Tat for 24 h or the Ras inhibitor FTS (Sigma-Aldrich, St. Louis, MO, USA) at 0, 5, 10, 20, 30, 40, or 50 *μ*mol/L for 12, 24, and 48 h. The cells were then incubated with 20 *μ*L 3-(4,5-dimethylthiazol-2-yl)-2,5-diphenyltetrazolium bromide solution (MTT, 5 mg/mL; Sigma-Aldrich) for another 4 h. Optical density was measured at 570 nm using a microwell plate reader (Thermo Scientific). Neither Tat at 1 *μ*g/mL nor FTS at 20 *μ*mol/L for 24 h had a significant effect on HBEC-5i cell viability.

### 2.3. Western Blot Analysis

Following treatment, cells were washed three times and lysed using radioimmunoprecipitation assay cell lysis buffer (Beyotime) containing protease inhibitor cocktail tablets (Beyotime). The lysates were collected and centrifuged at 12,000*g* for 15 min, and protein levels were quantified using a BCA Protein Assay Kit (Beyotime). Next, 30 *μ*g proteins were separated by SDS-PAGE and transferred onto polyvinylidene fluoride membranes (0.45 *μ*m; Millipore, Billerica, MA, USA). Membranes were blocked for 1 h with 5% fat-free milk at room temperature and then incubated with primary antibodies against ZO-1 (1 : 400, rabbit polyclonal; Abcam, Cambridge, UK), NEP (1 : 400, rabbit polyclonal; Abcam), and GAPDH (1 : 5000; Proteintech Group, Chicago, IL, USA) at 4°C overnight. The next day, the membranes were washed and incubated with IRDye 680 RD goat anti-rabbit immunoglobulin (Ig) G (1 : 10,000; LI-COR Biosciences, Lincoln, NE, USA) secondary antibody for 1 h at room temperature. The detected proteins were then visualized using an Odyssey Infrared Imaging System (LI-COR Biosciences). Band density was analysed by ImageJ software (National Institutes of Health, Bethesda, MD, USA), and signal density was calculated as the ratio of signal intensity to that of GAPDH.

### 2.4. Real-Time Reverse Transcription Polymerase Chain Reaction

After treatment, cells were harvested, total RNA was extracted using TRIzol reagent (TakaraBio, Dalian, Japan), and cDNA was generated from 1 *μ*g RNA using the Prime-Script RT reagent kit (Takara) according to the manufacturer's instructions. cDNA was used for quantitative RT-PCR using a Taq PCR Master Mix kit (Takara) and conducted on the StepOnePlus Real-Time PCR System (Applied Biosystems, Foster City, CA, USA) using RT Reaction Mix in a total volume of 20 *μ*L at 95°C for 30 s, followed by 95°C for 5 s, and 60°C for 30 s for 40 cycles. The primer sequences were as follows: ZO-1 (Takara): 5′-GACCAATAGCTGATGTTGCCAGAG-3′ and 5′-TGCAGGCGAATAATGCCAGA-3′; NEP (Takara): 5′-TAAGCAGCCTCAGCCGAACC-3′ and 5′-TTGACATAGTTTGCACAACGTCTCC-3′; and GAPDH (Takara): 5′-GCACCGTCAAGGCTGAGAAC-3′ and 5′-TGGTGAAGACGCCAGTGGA-3′. GAPDH was used to normalize target gene mRNA levels, which were analysed using the 2^−ΔΔCt^ method.

### 2.5. Immunofluorescence Microscopy

HBEC-5i cells were seeded onto gelatin-coated circular glass slips in 24-well plates and incubated for 24 h. After treatment, the cells were fixed with 4% paraformaldehyde (Solarbio, Beijing, China) on ice for 30 min and permeabilised with 0.1% TritonX-100 (Beyotime) for 10 min. They were then blocked with 3% bovine serum albumin (Sigma, St. Louis, MO, USA) for 1 h at room temperature before overnight incubation with a primary antibody against NEP (1 : 100, rabbit polyclonal; Abcam) at 4°C. After washing with phosphate-buffered saline (PBS), the slides were incubated with the Alexa Fluor-488 donkey anti-rabbit (1 : 500; Invitrogen, Thermo Fisher, Waltham, MA, USA) secondary antibody for 2 h at room temperature. After washing and staining with 4′,6-diamidino-2-phenylindole (Invitrogen), samples were visualized using a fluorescence microscope (OlympusBX53, Tokyo, Japan).

A*β* (1–40) HiLyte (a fluorescently labelled A*β*) was purchased from AnaSpec (San Jose, CA, USA). A*β* (1–40) HiLyte was first dissolved in PBS and then diluted in cell culture medium as suggested by the manufacturer. Cells were treated with A*β* (1–40) HiLyte at the concentration of 1 *μ*mol/L for 10 min in serum-free medium as previously described [1]. The cells were then fixed, washed, and mounted. The fluorescence signal from A*β* (1–40) HiLyte was directly acquired with a fluorescence microscope (OlympusBX53). Both acquisition and quantification were performed using Olympus cellSens Dimension software.

### 2.6. A*β* (1–40) Transport across HBEC-5i Monolayers

Confluent HBEC-5i cells cultured on Transwell filter inserts (pore size 0.4 *μ*m, 24-well cell culture plate; Corning, Corning, NY, USA) were pretreated with 5 *μ*mol/L FTS for 3 h and exposed to 1 *μ*g/mL HIV-1 Tat for 24 h. For the last 20 min of HIV-1 Tat exposure, 1 *μ*mol/L A*β* (1–40) HiLyte was added to the upper chamber incubated at 37°C in the dark. The fluorescence signal from A*β* (1–40) HiLyte as the indicator of transendothelial A*β* transfer was measured using a multidetection microplate reader (Bio-Rad, Hercules, CA, USA) in the lower chamber at 503 nm (excitation) and 528 nm (emission).

### 2.7. Detection of Intracellular ROS

Intracellular ROS levels were quantified using the Reactive Oxygen Species Assay Kit (Beyotime). Cells were washed with PBS, and then, a final concentration of 10 mmol/L DCFH-DA was added for 30 min incubation at 37°C in the dark. After washing with PBS, the stained cells in the 24-well plate were visualized by inverted fluorescence microscopy (Nikon, Tokyo, Japan). Relative levels of fluorescence in cells were measured with a multidetection microplate reader (Bio-Rad) at 488 nm (excitation) and 525 nm (emission). Intracellular ROS level was expressed as the percentage of the control group.

### 2.8. Statistical Analysis

The data are shown as means ± standard deviations. Data were analysed using SPSS version 17.0 (SPSS, Chicago, IL, USA). One-way ANOVA was used to compare responses between groups. Differences were considered statistically significant at *p* < 0.05.

## 3. Results

### 3.1. Cell Viability

**Figure 1:**
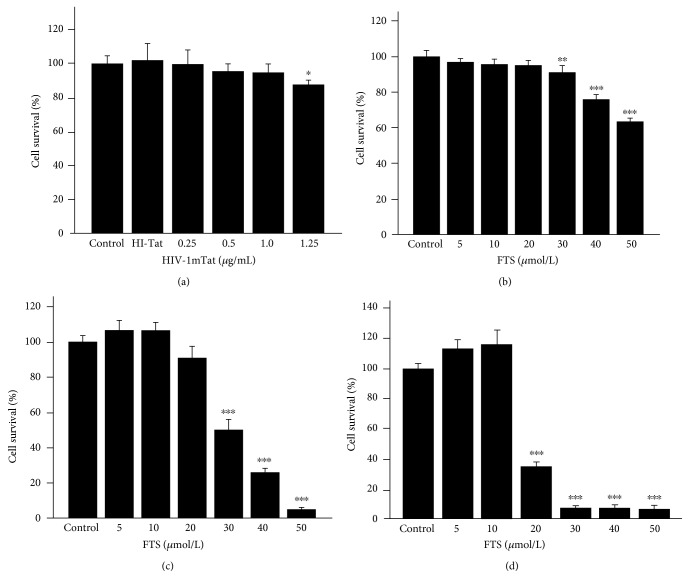
Cell viability assay. HBEC-5i cells were treated with HIV-1 Tat at 0, 0.25, 0.5, 1, or 1.25 *μ*g/mL and heat-inactivated Tat for 24 h (a) or FTS at 0, 5, 10, 20, 30, 40, or 50 *μ*mol/L for 12, 24, or 48 h (b, c, d). Cell viability was not affected by 1 *μ*g/mL HIV-1 Tat or 20 *μ*mol/L FTS for 24 h. Results are expressed as means ± standard error of the mean (*n* = 5). ^∗^*p* < 0.05, ^∗∗^*p* < 0.01, and ^∗∗∗^*p* < 0.001 versus that in the control.

HBEC-5i viability was tested with MTT assays. Neither HIV-1 Tat at 1 *μ*g/mL nor FTS at 20 *μ*mol/L for 24 h had a significant effect on HBEC-5i cell viability (Figure 1).

### 3.2. Ras Signaling Is Involved in HIV-1 Tat-Induced Disruption of ZO-1

**Figure 2:**
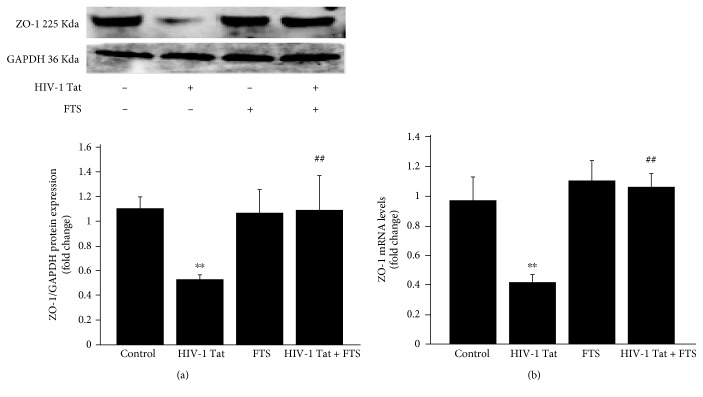
Role of Ras signaling in HIV-1 Tat-induced disruption of ZO-1. Before HIV-1 Tat exposure, HBEC-5i cells were pretreated with 5 *μ*mol/L FTS for 3 h. FTS remained in the culture medium during Tat exposure. The time of Tat exposure was 24 h for western blotting (a) and 12 h for RT-PCR (b). HIV-1 Tat exposure induced decreased ZO-1 protein and mRNA levels in HBEC-5i cells. Following cotreatment with FTS and HIV-1 Tat, ZO-1 protein and mRNA levels were significantly increased compared with those in the HIV-1 Tat group. Data are shown as means ± standard error of the mean (*n* = 3 for (a), *n* = 5 for (b)). ^∗∗^*p* < 0.01 versus control; ^##^*p* < 0.01 versus HIV-1 Tat.

To observe the effects of HIV-1 Tat on the TJ protein ZO-1, HBEC-5i cells were exposed to 1 *μ*g/mL HIV-1 Tat for 24 h (for western blotting) or 12 h (for real-time RT-PCR). Both protein and mRNA levels of ZO-1 were significantly lower in the HIV-1 Tat group compared to the control group (Figures 2(a) and 2(b)).

To determine if Ras signaling is involved in HIV-1 Tat-induced ZO-1 downregulation, HBEC-5i cells were pretreated for 3 h with 5 *μ*mol/L FTS, followed by cotreatment with FTS and 1 *μ*g/mL HIV-1 Tat for 24 or 12 h. ZO-1 protein and mRNA levels were significantly increased following coexposure to FTS and HIV-1 Tat compared with the HIV-1 Tat group (Figures 2(a) and 2(b)).

### 3.3. Ras Signaling Is Involved in HIV-1 Tat-Induced Regulation of NEP Expression

**Figure 3:**
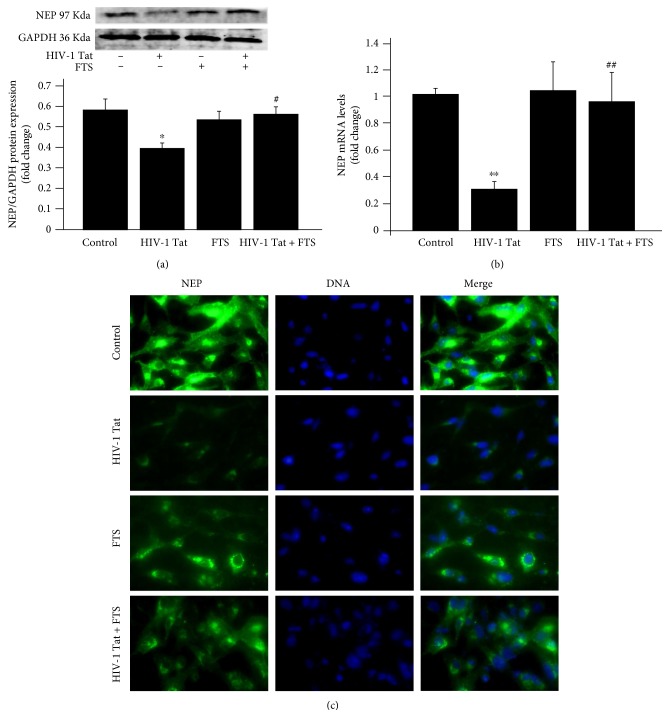
Role of Ras signaling in HIV-1 Tat-induced changes in NEP. Exposure to HIV-1 Tat resulted in marked decreases in NEP protein (a) and mRNA (b) and weaker immunoreactivity (c) compared with the control group. Prior to HIV-1 Tat exposure, cells were pretreated with 5 *μ*mol/L FTS for 3 h with FTS in the culture medium. NEP protein and mRNA levels and immunoreactivity significantly increased with coexposure to FTS and HIV-1 Tat compared with the HIV-1 Tat group. Data are expressed as means ± standard error of the mean (*n* = 3 for (a), *n* = 5 for (b)). ^∗^*p* < 0.05, ^∗∗^*p* < 0.01 versus that in the control; ^#^*p* < 0.05, ^##^*p* < 0.01 versus that in HIV-1 Tat.

To investigate whether HIV-1 Tat could affect the expression of NEP in HBEC-5i cells, its expression was detected with western blotting, real-time RT-PCR, and immunofluorescence. Treatment with 1 *μ*g/mL HIV-1 Tat for 24 h (for western blotting and immunofluorescence) or 12 h (for real-time RT-PCR) significantly downregulated NEP protein and mRNA levels (Figures 3(a) and 3(b)) and resulted in markedly weaker NEP immunoreactivity compared with the control group (Figure 3(c)).

To evaluate whether Ras signaling affects HIV-1 Tat-induced changes in NEP expression, HBEC-5i cells were pretreated with 5 *μ*mol/L FTS for 3 h and then cotreated with FTS and HIV-1 Tat for 24 or 12 h. FTS increased NEP protein levels (Figure 3(a)). These results were consistent with the NEP mRNA levels in the groups cotreated with FTS and HIV-1 Tat versus the HIV-1 Tat group (Figure 3(b)), and markedly stronger NEP immunoreactivity was noted in the FTS-treated cells compared with the HIV-1 Tat group (Figure 3(c)).

### 3.4. Ras Signaling Is Involved in HIV-1 Tat-Induced Accumulation of Exogenous A*β*

**Figure 4:**
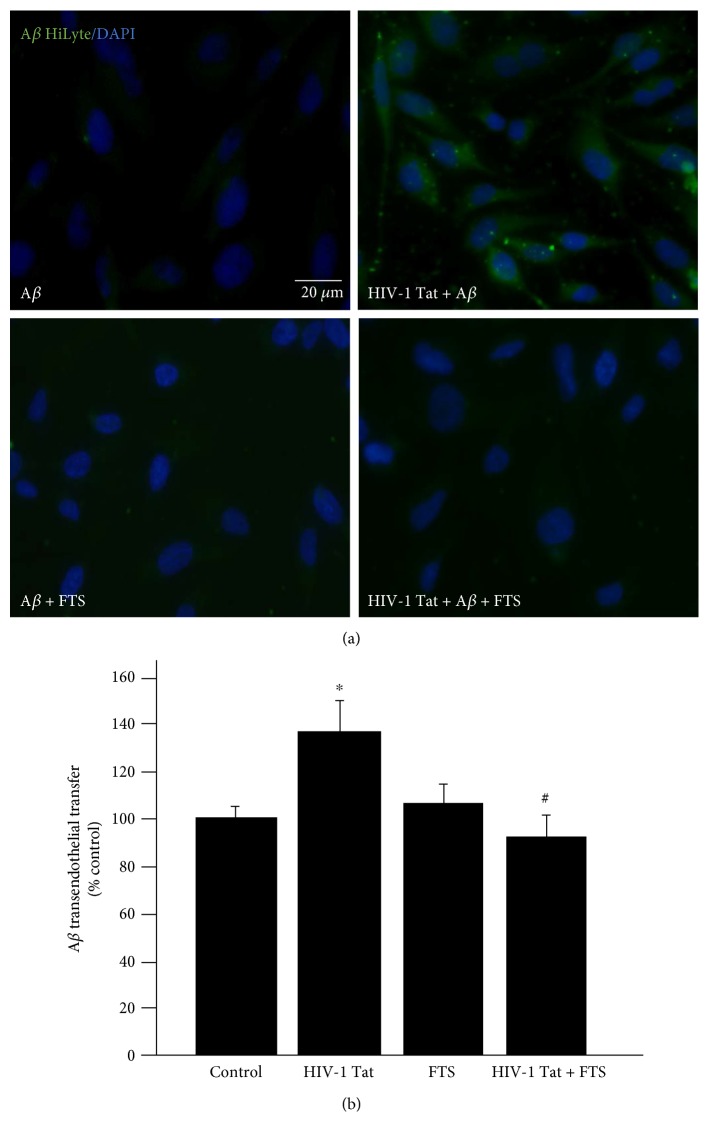
Role of Ras signaling in HIV-1 Tat-induced accumulation of exogenous A*β* in HBEC-5i. HIV-1 Tat exposure markedly increased the accumulation of A*β* (1–40) HiLyte in HBEC-5i. Pretreatment with 5 *μ*mol/L FTS for 3 h followed by coexposure to HIV-1 Tat and FTS significantly decreased A*β* (1–40) HiLyte accumulation (a). FTS inhibited HIV-1 Tat-induced transendothelial transfer of A*β*. Confluent HBEC-5i cells in Transwell filter inserts were exposed to 1 *μ*mol/L A*β* (1–40) HiLyte for the last 20 min of HIV-1 Tat exposure in the upper chamber, mimicking the blood side of the BBB. The fluorescence signal of A*β* (1–40) HiLyte was measured in the lower chamber, equivalent to the brain side of the BBB (b). Data are expressed as means ± standard error of the mean (*n* = 4). ^∗^*p* < 0.05 versus that in the control; ^#^*p* < 0.05 versus that in HIV-1 Tat.

To observe the combined effects of HIV-1 Tat and exogenous A*β* on intracellular A*β* levels, HBEC-5i cells were exposed to 1 *μ*g/mL HIV-1 Tat for 24 h. For the last 10 min of HIV-1 Tat treatment, A*β* (1–40) HiLyte was added to the culture medium. As shown in Figure 4(a), green fluorescence from A*β* (1–40) HiLyte was significantly increased in the presence of HIV-1 Tat compared with the control group treated with A*β* (1–40) HiLyte alone.

To explore whether Ras signaling is involved in HIV-1 Tat-induced effects of exogenous A*β*, HBEC-5i cells were pretreated with 5 *μ*mol/L FTS for 3 h followed by coexposure to FTS and 1 *μ*g/mL HIV-1 Tat for 24 h. As shown in Figure 4(a), FTS pretreatment prior to coexposure to HIV-1 Tat and FTS markedly decreased HIV-1 Tat-induced accumulation of A*β* (1–40) HiLyte.

To evaluate the effect of HIV-1 Tat and/or FTS on A*β* transfer across HBEC-5i monolayers, HBEC-5i cells were exposed to 1 *μ*mol/L A*β* (1–40) HiLyte during HIV-1 Tat treatment in a Transwell model system. This led to significant transendothelial A*β* transfer, which was significantly attenuated by a 3 h pretreatment with 5 *μ*mol/L FTS before cotreatment with HIV-1 Tat and FTS (Figure 4(b)).

### 3.5. ROS Production Induced by HIV-1 Tat in HBEC-5i Is Attenuated by Ras Inhibition

**Figure 5:**
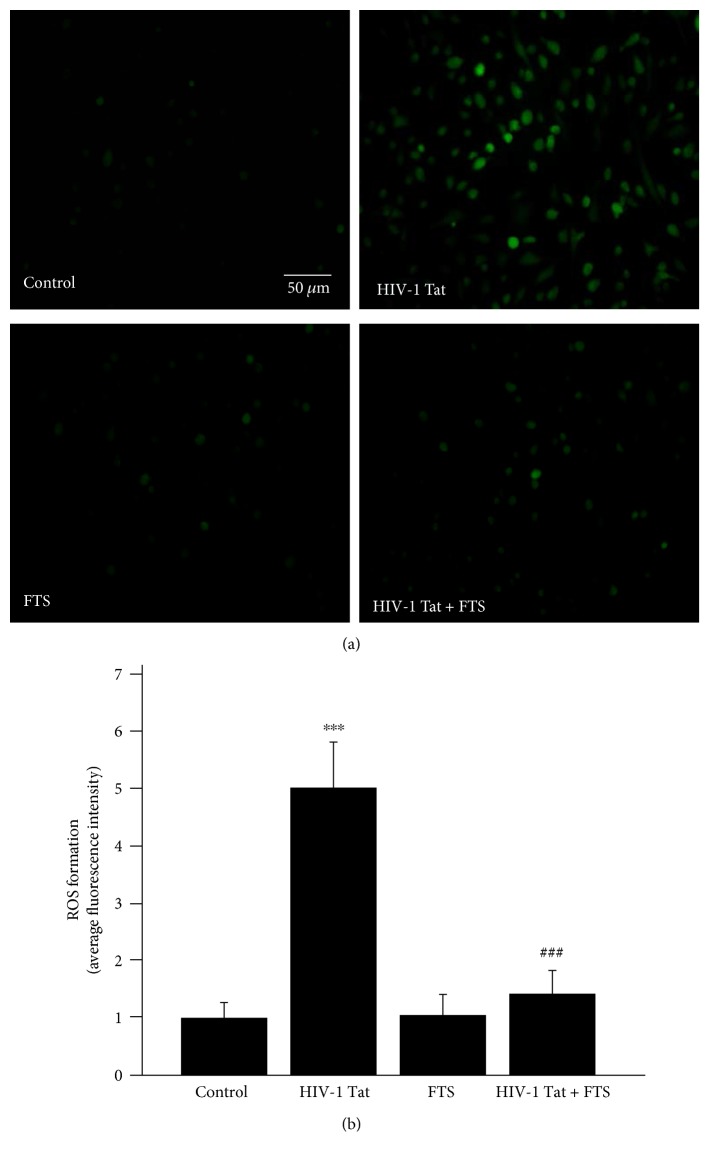
Role of Ras signaling in HIV-1 Tat-induced changes in intracellular ROS levels. Exposure to HIV-1 Tat for 24 h significantly increased intracellular ROS levels in HBEC-5i cells. Pretreatment with 5 *μ*mol/L FTS and then coexposure to HIV-1 Tat and FTS markedly attenuated HIV-1 Tat-induced intracellular ROS production. Results are shown as means ± standard error of the mean (*n* = 4). ^∗∗∗^*p* < 0.01 versus that in the control; ^###^*p* < 0.01 versus that in HIV-1 Tat.

To observe the effects of HIV-1 Tat on intracellular ROS levels, overall intracellular ROS production in HBEC-5i cells was assessed with DCFDA staining. Exposure to HIV-1 Tat significantly increased intracellular ROS levels, but this was ameliorated by pretreatment with 5 *μ*mol/L FTS (Figure 5).

## 4. Discussion

HIV-1 Tat protein is regarded a pathogenic factor in HIV-associated neurocognitive disorders (HAND) [26–29]; however, how HIV-1 Tat induces the development of AD-like pathology in HIV-1-infected patients is not fully understood. HAND in older patients seems to be linked to early beta-amyloidosis [3, 8]. HIV-1 Tat is reportedly both neuroexcitatory and neurotoxic [30] and can enhance the adhesion of monocytes and T cells to the endothelium both in vivo and in vitro [31]. A previous study found that two mechanisms underlying HIV-1 Tat-induced BBB destruction were elevated cellular oxidative stress and stimulated inflammatory responses [32]. Our previous study reported that HIV-1 Tat disrupted the TJ protein occludin, downregulated low-density lipoprotein receptor-related protein 1 expression, and even upregulated the expression of a receptor for advanced glycation end products, but all of these outcomes were attenuated by inhibiting Rho/ROCK signaling [33]. A functionally impaired BBB with deficient A*β* clearance could lead to brain A*β* accumulation [11]; therefore, the BBB is critical for preventing A*β* deposition in the HIV-infected brain.

HIV-1 Tat activates Ras and then leads to ERK phosphorylation in endothelial cells [34]. Ras signaling induces ROS generation via the downstream effector MEK/ERK [20], and oxidative stress may be an early step in Tat-induced neurotoxicity [35]. The elements of the Ras signaling cascade are involved in proinflammatory signal transduction [36]. Ras proteins can also activate the immune system [23]. Inhibition of the Ras pathway by FTS was previously found to protect brain endothelial cells from Tat-induced disruption of TJ proteins [25]. ZO-1 acts as a scaffold protein linking occludin to the actin cytoskeleton, and it plays a critical role in maintaining BBB resistance and permeability [14]. Knocking down ZO-1 with siRNA leads to barrier hyperpermeability [36]. HBEC-5i cells have major characteristics of cerebral endothelial cells, such as high transendothelial electrical resistance (TEER) and low permeability [37]. A previous study demonstrated that exposure to HIV-1 Tat increased permeability and decreased TEER of brain microvascular endothelial cells [38]. In accordance with the earlier study [25], in the present study, HIV-1 Tat-mediated downregulation of ZO-1 protein and mRNA levels was attenuated by inhibiting Ras signaling with FTS. Collectively, these findings indicate that FTS protected against HIV-1 Tat-induced BBB dysfunction partly by inhibiting Ras signaling.

NEP is considered a critical A*β*-degrading enzyme in the brain [39] that can degrade both extracellular and intracellular A*β* [4]. Levels were increased obviously in the brains of NEP knockout mice [40]. However, the mechanisms of Tat-induced inhibition of NEP are relatively unknown. A previous study reported that the regulation of NEP activity may involve MEK/ERK signaling; specifically, MEK inhibition increases NEP activity [41]. In addition, increasing intracellular ROS production may also downregulate NEP expression in vascular endothelial cells [42]. In the present study, HIV-1 Tat treatment reduced NEP protein and mRNA levels; however, these effects were diminished by FTS treatment, and the effect of HIV-1 Tat on weaker NEP immunoreactivity was reversed by FTS. Exposure to HIV-1 Tat also significantly elevated intracellular ROS levels, which were decreased by FTS. These results suggest that FTS may partly protect against HIV-1 Tat-induced inhibition of NEP by ameliorating oxidative stress (decreased ROS formation) and inhibiting Ras cascades.

A previous study demonstrated that Tat clade-B may possess a unique ability to directly stimulate both A*β* formation and cell-bound A*β* accumulation [5]. Exposure to HIV-1 also increases A*β* transendothelial transfer [1]. A*β* is a proinflammatory factor that may induce chronic neuroinflammatory responses and damage the endothelium [8]. A*β* toxicity in the brain vasculature seems to induce ROS production [1]. In accordance with the earlier findings [3], we showed that treatment with exogenous A*β* (1–40) HiLyte increased intracellular A*β* levels, and this effect was further enhanced by the presence of HIV-1 Tat. Notably, inhibition of Ras by FTS effectively prevented HIV-1 Tat-mediated A*β* accumulation. In addition, Tat treatment enhanced transendothelial transfer of A*β* (1–40) HiLyte; this finding is in conformity with the earlier findings [8]; however, this effect was attenuated by Ras inhibition. These data suggest that inhibiting Ras may attenuate HIV-1 Tat-induced A*β* accumulation and transendothelial transfer.

In summary, inhibition of Ras signaling in HBEC-5i cells significantly attenuated HIV-1 Tat-induced disruption of ZO-1 and NEP, decreased HIV-1 Tat-induced intracellular ROS formation, and protected against HIV-1 Tat-induced A*β* accumulation. These findings clarify the possible mechanisms involved in HIV-1-induced A*β* accumulation in the brain. They also indicate a potential protective effect of FTS on HIV-1 Tat-mediated BBB dysfunction and A*β* accumulation. Targeting the Ras signaling pathway may be a promising approach for HAND.
